# ^1^H, ^15^N and ^13^C backbone resonance assignments of flap endonuclease from *Plasmodium falciparum*

**DOI:** 10.1007/s12104-025-10241-6

**Published:** 2025-08-09

**Authors:** Rodolpho do Aido-Machado, Nicola J. Baxter, Michelle L. Rowe, Manoj B. Pohare, Srdjan Vitovski, Jon R. Sayers, Jonathan P. Waltho

**Affiliations:** 1 Division of Clinical Medicine, School of Medicine and Population Health, The University of Sheffield, Medical School, Beech Hill Road, Sheffield, S10 2RX UK; 2 School of Biosciences, The University of Sheffield, Firth Court, Western Bank, Sheffield, S10 2TN UK; 3 Manchester Institute of Biotechnology, School of Chemistry, The University of Manchester, 131 Princess Street, Manchester, M1 7DN UK

**Keywords:** Flap endonuclease, Backbone resonance assignment, Transverse relaxation optimised spectroscopy, *Plasmodium falciparum*, Early-stage drug discovery

## Abstract

Flap endonuclease (FEN) enzymes are a group of metallonucleases that have essential roles in DNA repair and the maintenance of genomic resilience. They catalyse hydrolytic cleavage of a phosphodiester bond to remove 5′-flaps present on double-stranded DNA molecules formed during DNA replication. FEN locates a target scissile bond through the structural recognition of a pronounced bend in the double-stranded DNA substrate along with the presence of both a 5′-flap and a 1-nucleotide 3′-flap. FEN enzymes share a common structural architecture and are functionally conserved across all living organisms. In this work, we report the ^1^H, ^15^N and ^13^C backbone resonance assignments of residues 2–349 of FEN from *Plasmodium falciparum* (*Pf*FEN349) in its substrate-free state. Using heteronuclear multidimensional NMR spectroscopy, 90% of all backbone resonances of *Pf*FEN349 were assigned, with 298 backbone amide resonances out of 337 theoretically-detectible resonances (which exclude 10 prolines and the N-terminal glycine) identified in the ^1^H–^15^ N TROSY spectrum. Prediction of solution secondary structure content from a chemical shift analysis using the TALOS-N webserver is mostly in good agreement with an AlphaFold model of *Pf*FEN349. The reported assignments provide a pathway for drug discovery as *Pf*FEN349 is a potential target for the development of new inhibitors that could be utilised to control the incidence of malaria across the globe.

**Supplementary Information:**

The online version contains supplementary material available at 10.1007/s12104-025-10241-6.

## Biological context

Flap endonuclease (FEN) enzymes (EC 3.1.99.B1), also known as 5′ nucleases or 5′-3′ exonucleases, are a group of metallonucleases that catalyse hydrolytic cleavage of a phosphodiester bond to remove 5′-flaps present on double-stranded DNA molecules. These 5′-flaps are temporary single-stranded overhang structures generated during DNA replication through the extension of short RNA primers while creating Okazaki fragments on the lagging DNA strand. A 5′-flap structure is formed as the 3′ end of the lagging strand extends towards the 5′ end of the RNA primer that started the previous Okazaki fragment, de-annealing it through strand-displacement synthesis (Burgers 2009). FEN is nucleotide sequence independent and locates a target scissile bond through the precise recognition of structural features that include a pronounced bending (~ 100°) of the nicked double-stranded DNA substrate and the presence of both a 5′-flap and a 1-nucleotide 3′-flap positioned adjacently. FEN has a crucial role in long-patch base excision repair (Klungland and Lindahl 1997), which is a cellular mechanism employed to remove short sections. (2–10 nucleotides) of damaged DNA during the cell cycle. In addition, FEN can also perform exonucleolytic activity (Murante et al. 1994; Williams et al. 2007) and has been reported to have gap specific endonuclease activity (Zheng et al. 2005). Together, these roles enable FEN to participate in several DNA metabolic pathways, including stalled replication fork rescue, telomere maintenance, resolution of tri-nucleotide repeat sequence-derived secondary structures and apoptotic DNA fragmentation (Balakrishnan and Bambara 2013).

FEN enzymes are functionally conserved and occur across all living organisms, from viruses and archaebacteria to plants, fungi and mammals (Shen et al. 1998). They are crucial for cell survival in prokaryotes (Bayliss et al. 2005; Díaz et al. 1992; Fukushima et al. 2007; Lowder and Simmons 2023). Many eubacteria have more than one homologue (Allen et al. 2009; Fukushima et al. 2007), and this redundancy, along with at least one functional homologue being required for cellular viability, points to their essential roles in the maintenance of genomic integrity. FEN enzymes are also essential in eukaryotes, where their activity can be modulated by interaction with over 30 known protein partners or through posttranslational modifications such as acetylation, methylation, phosphorylation, SUMOylation and ubiquitination (Zheng et al. 2011). Furthermore, reduced FEN expression in haplo-insufficient mouse cells results in genome instability and rapid tumour progression, while FEN-knockout mice do not develop due to embryonic lethality (Kucherlapati et al. 2002; Larsen et al. 2003). Notably, human flap endonuclease 1 (hFEN1) plays a significant role in cancer development and its overexpression is observed in various cancer types. An abundance of hFEN1 leads to increased mutation within the genome that likely contributes to cancer formation and evolution (Becker et al. 2018). Such activity positions hFEN1 as an important potential therapeutic target for cancer treatments.

**Fig. 1:**
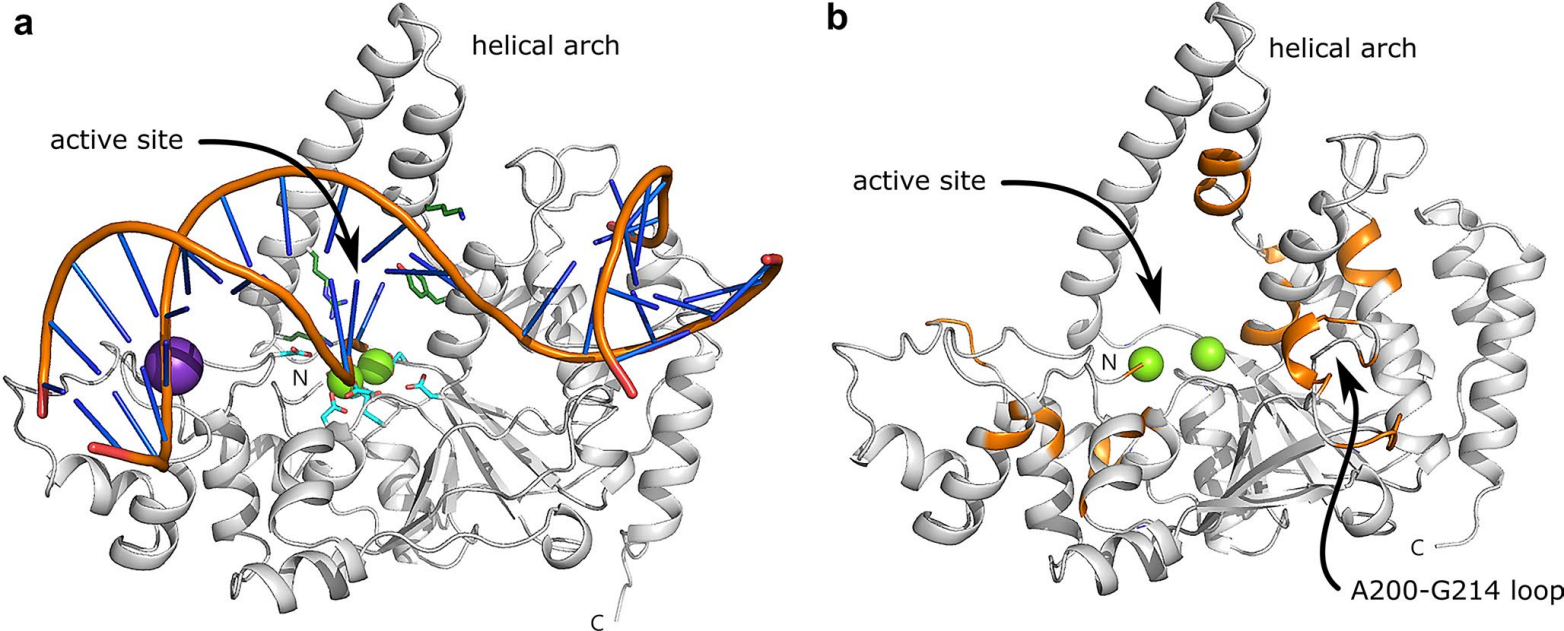
Structural comparison of DNA-bound hFEN1 and the substrate-free AlphaFold model of *Pf*FEN349. (**a**) Cartoon representation of DNA-bound hFEN1. The crystal structure of hFEN1 (PDB 3Q8K, Tsutakawa et al. 2011) is illustrated with the protein backbone coloured grey and the bound DNA product coloured orange (deoxyribose-phosphodiester backbone) and blue (nucleobases). Positions of the active site and helical arch are labelled. The catalytic ions within the active site are shown as green spheres (samarium^III^ ions were used as a surrogate for magnesium ions in this structure), and a potassium ion that mediates electrostatic interactions with DNA is shown as a purple sphere. The catalytic ions are coordinated by numerous aspartate and glutamate residues (Asp34, Asp86, Glu158, Glu160, Asp179, Asp181 and Asp233) and are indicated as cyan sticks. Key catalytic residues (Tyr40, Lys93, Arg100 and Lys128) that interact with DNA within the active site are represented as dark green sticks. (**b**) Cartoon representation of an AlphaFold model of *Pf*FEN349. The protein backbone is coloured grey and unassigned residues in the ^1^H–^15^ N TROSY spectrum of *Pf*FEN349 are coloured orange. The unassigned residues, mostly owing to peak attenuation through conformational exchange dynamics occurring on the millisecond timescale, broadly locate to regions of the enzyme important for binding the DNA substrate and catalysing the chemical step. The catalytic magnesium ions are shown as green spheres and the position of the 15-residue Ala200–Gly214 loop is highlighted. Structural figures and superpositions were created using PyMOL (The PyMOL Molecular Graphics System version 1.8/2.2. Schrodinger LLC)

Crystal structures of FEN from various organisms reveal a shared common architecture. A central β-sheet (typically comprising seven strands) is flanked by α-helical bundles, which together form a saddle-like structure (Fig. 1A). The DNA substrate is accommodated along the length of this saddle, with the phosphodiester backbone groups coordinated by arginine and lysine residues (Garforth et al. 1999; Patel et al. 2002). Projecting from the saddle above the active site is a feature known as the helical arch and its conformation is the most variable among the crystal structures reported (AlMalki et al. 2016; Bennet et al. 2018). At the conformational extremes, the helical arch either is composed of two fully structured α-helices or adopts a disordered flexible loop (Mueser et al. 1996; Anstey-Gilbert et al. 2013; Ghosh et al. 2020). However, in most crystal structures, this region has both structured and disordered segments. NMR analysis of hFEN1 indicates that in solution, the central portion of the helical arch forms two short α-helices while residues located close to the saddle are unassignable due to intermediate exchange on the millisecond timescale (Bennet et al. 2018). Addition of a DNA substrate increases the number of residues experiencing such exchange. This observation points to inherent dynamics occurring in this region that potentially allows the 5′-flap to be threaded through the helical arch where it becomes coordinated by conserved arginine and lysine residues. Such positioning allows precise engagement of the scissile phosphodiester bond within the active site (Ceska et al. 1996; Dervan et al. 2002; Tsutakawa et al. 2011; AlMalki et al. 2016). Additionally, two essential catalytic magnesium ions (Fig. 1A) that are chelated by conserved carboxylate groups (mostly aspartic acid residues) promote the development of a nucleophilic hydroxyl ion prior to catalysis. In vitro studies show that these catalytic magnesium ions can be replaced with a range of surrogate divalent metal ions (calcium, cobalt, copper, iron, manganese, nickel and zinc) thereby modulating activity (Garforth et al. 2001; Feng et al. 2004; Syson et al. 2008; Ghosh et al. 2020).

The genome of unicellular protozoan parasite *Plasmodium falciparum*, which is the causative agent of malaria, encodes a FEN *(Pf*FEN) (NIH XP_001351399.1: flap endonuclease 1 *Plasmodium falciparum* 3D7). *Pf*FEN performs crucial roles in cellular DNA replication and repair, with damaged DNA being processed primarily through the long-patch base excision repair system to maintain genome fidelity (Haltiwanger et al. 2000). *Pf*FEN consists of 672 residues and has a molecular weight of 76.7 kDa. It features an N-terminal nuclease domain (350 residues) and a C-terminal domain (322 residues) comprised of low complexity sequence rich in asparagine (23%), lysine (15%), aspartate (12%) and serine (11%) residues. The AlphaFold structural model of *Pf*FEN (Varadi et al. 2022, 2024) reveals that the C-terminal domain is natively disordered and has no discernible secondary structure. Intrinsically-unstructured proteins or domains are abundant within the *Plasmodium falciparum* proteome and may have roles to facilitate parasite survival and disease progression through their ability to allow promiscuous binding interactions with a range of host molecules (Feng et al. 2006; Casta et al. 2008). The AlphaFold model of the nuclease domain of *Pf*FEN (Fig. 1B) overlays closely with both substrate-free hFEN1 (PDB 5ZOD, Xu et al. 2018; non-H atom RMSD = 1.90 Å) and DNA-bound hFEN1 (PDB 3Q8K, Tsutakawa et al. 2011; non-H atom RMSD = 1.77 Å), despite sharing 47% sequence identity with hFEN1. An additional 15-residue loop in *Pf*FEN, between Ala200 and Gly214 (ATSNQNKNKNNSKRG), may assist with DNA coordination due to the presence of an arginine and three lysine residues (Fig. 1B). Given its essential role in cellular viability, *Pf*FEN provides an attractive target for the development of new inhibitors that could be utilised to control the incidence of malaria across the globe. The recent escalation of drug resistance to previously established treatments threatens to undermine the management of this deadly disease, particularly in countries where it is endemic. As a first step on the pathway to drug discovery, the solution behaviour of *Pf*FEN was explored using NMR spectroscopy with the purpose of generating chemical shift assignments that could be used for ligand screening. In this work, we report the ^1^H, ^15^N and ^13^C backbone resonance assignments of the N-terminal nuclease domain (residues 2–349) of *Pf*FEN (*Pf*FEN349) in its substrate-free state.

## Methods and experiments

### Expression and purification of *Pf*FEN405

Initially, a synthetic codon-optimised gene encoding residues 2–405 from *Plasmodium falciparum* FEN (*Pf*FEN405) was purchased from Eurofins Genomics and inserted into the pT5P plasmid (Brudenell et al. 2024) using EcoRI and HindIII restriction enzymes. *Pf*FEN405 (46.1 kDa) comprises the N-terminal nuclease domain, together with an additional 56 residues from the C-terminal domain. The plasmid was transformed into *Escherichia coli* strain BL21 (TransGen Biotech) using carbenicillin selection (100 mg·L^− 1^) and ^15^N-labelled *Pf*FEN405 was expressed in defined isotopically labelled minimal media with ^15^NH_4_Cl (1 g·L^− 1^) as the sole nitrogen source (Reed et al. 2003). The cells were grown at 37 °C with shaking until OD_600nm_ = 0.7, at which point they were cooled to 20 °C and induced with isopropyl β-D-1-thiogalactopyranoside (IPTG) to a final concentration of 0.5 mM. The cultures were incubated with shaking for a further 16 h and were harvested by centrifugation at 7000 rpm for 20 min at 4 °C in a Sorvall Lynx 6000 centrifuge (rotor F9-6 × 1000 LEX). The cell pellet was resuspended with 5 mL per gram of cells using cold lysis buffer (25 mM HEPES pH 7.4, 100 mM NaCl, 5 mM EDTA, 5 mM DTT and 5% glycerol). This resuspension was then supplemented with deoxycholic acid sodium salt (2.5 mg per gram of cells), 4-(2-aminoethyl)benzenesulfonyl fluoride hydrochloride (1.2 mg per gram of cells) and lysozyme (1 mg per gram of cells) and incubated at room temperature on a roller for 1 h. The viscous cell suspension was lysed on ice by sonication for 10 cycles consisting of 10 s pulsation followed by a 40 s cooling interval, with gentle mixing in between. The cell lysate was then separated by ultracentrifugation at 19000 rpm for 30 min at 4 °C in a Sorvall Lynx 6000 centrifuge (rotor F21-8 × 50y). Ammonium sulphate was added to the supernatant to a final concentration of 0.5 M and after its complete solubilisation, 70 µL per mL of solution of 5% polyethylenimine (PEI-HCl, pH 8) was added and mixed well. This mixture was incubated at 4 °C for 30 min and then centrifuged at 19000 rpm at 4 °C for 30 min. The pellet was discarded and ammonium sulphate was added slowly with gentle agitation until complete solubilisation had occurred to give a final concentration of 3.5 M. This solution was incubated for 1 h at 4 °C to precipitate *Pf*FEN405, which was pelleted as before by centrifugation. The pellet was resuspended carefully in Buffer A (25 mM HEPES pH 7.4, 5 mM EDTA, 5 mM DTT and 5% glycerol) and dialysed at 4 °C overnight against 1 L of the same buffer. The solution was centrifuged at 19000 rpm for 30 min at 4 °C and the supernatant was filtered through a 0.22 μm syringe. Column chromatography was performed using an ÄKTA Pure purification system (Cytiva). The filtered protein solution was loaded onto a HiPrep SP FF 16/10 ion-exchange column (Cytiva) that had been washed previously with 1 column volume (CV) of filtered and degassed Buffer B (25 mM HEPES pH 7.4, 5 mM EDTA, 5 mM DTT, 5% glycerol and 2 M NaCl) and equilibrated with 3 CV of filtered and degassed Buffer A (25 mM HEPES pH 7.4, 5 mM EDTA, 5 mM DTT and 5% glycerol). Proteins bound to the HiPrep SP FF 16/10 ion-exchange column were eluted with a gradient of 0 to 35% Buffer B. Fractions were checked for the presence of *Pf*FEN405 by SDS-PAGE, and were pooled together and diluted 5-fold in Buffer A. The protein sample was then loaded onto a HiTrap Heparin HP affinity column (Cytiva) that had been washed previously with 1 CV of filtered and degassed Buffer B and equilibrated with 3 CV of filtered and degassed Buffer A. Proteins bound to the HiTrap Heparin HP affinity column were eluted with a gradient of 0 to 35% Buffer B. Fractions were checked for the presence of *Pf*FEN405 by SDS-PAGE, and were pooled together and concentrated by Amicon Ultra Centrifugal Filters (10 kDa MWCO, Merck Millipore). The protein sample was loaded onto a prepacked HiLoad 16/600 Superdex 200 pg size-exclusion column (Cytiva) equilibrated with 1.5 CV of filtered and degassed Buffer A supplemented with 400 mM NaCl. Fractions containing *Pf*FEN405 were checked for purity by SDS-PAGE, and were pooled together and flash-frozen in liquid nitrogen prior to storage at − 80 °C. NMR samples were prepared by buffer exchanging and concentrating thawed *Pf*FEN405 into NMR buffer (20 mM sodium phosphate pH 7.4, 30 mM KCl, 10 mM MgCl_2_, 0.1 mM EDTA, 2 mM NaN_3_ and 2 mM tris(2-chloroethyl) phosphate (TCEP) using Amicon Ultra Centrifugal Filters (10 kDa MWCO, Merck Millipore). The nuclease activity of *Pf*FEN405 was confirmed by FRET assay (AlMalki et al. 2016). Protein concentrations were estimated by absorbance at 280 nm (extinction coefficient = 21890 M^− 1^·cm^− 1^). All reagents were of analytical grade and were purchased from Sigma-Aldrich (UK).

### NMR spectroscopy of *Pf*FEN405

A 5-mm NMR tube was loaded with 0.2 mM ^15^N-labelled *Pf*FEN405 in NMR buffer supplemented with ^2^H_2_O (10% v/v) for the deuterium lock and 1 mM trimethylsilyl propanoic acid (TSP) as a chemical shift reference. A 2D ^1^H–^15^ N TROSY experiment was recorded at 298 K using an 800 MHz Bruker Neo 4-channel spectrometer fitted with a 5-mm TCI cryoprobe equipped with z-axis gradients and running TopSpin software version 4.0.5. The ^1^H–^15^N TROSY spectrum showed a considerable number of peaks and their distribution is consistent with the N-terminal nuclease domain being folded under the conditions of the NMR experiment (Supplementary Fig. 1A). A large number of overlapped peaks were also present in the centre of the spectrum, resonating at random coil chemical shifts. These observations support the AlphaFold structural model of *Pf*FEN, where the nuclease domain adopts a folded conformation while residues of the C-terminal domain remain disordered. Such severe crowding of peaks in the ^1^H–^15^ N TROSY spectrum of *Pf*FEN405 would likely hamper the procedure for spectral assignment. Therefore, through combined use of the NMR data and the AlphaFold model, an alternative construct was designed that only consisted of the structured nuclease domain of *Pf*FEN.

### Expression and purification of *Pf*FEN349

A construct containing residues 2–349 from *Plasmodium falciparum* FEN (*Pf*FEN349) was generated in the pT5P plasmid and confirmed by DNA sequencing. *Pf*FEN349 (39.6 kDa) comprises just the N-terminal nuclease domain and lacks any residues from the unstructured C-terminal domain. The plasmid was transformed into *Escherichia coli* strain BL21 (TransGen Biotech) using carbenicillin selection (100 mg·L^− 1^). ^15^N-labelled *Pf*FEN349 was expressed in defined isotopically labelled minimal media with ^15^NH_4_Cl (1 g·L^− 1^) as the sole nitrogen source. ^2^H,^15^N,^13^C-labelled *Pf*FEN349 was expressed in defined isotopically labelled minimal media with protiated solutions replaced by deuterated solutions, and with ^15^NH_4_Cl (1 g·L^− 1^) and ^13^C_6_,^2^H_7_-labelled glucose (3 g·L^− 1^) as the sole nitrogen and carbon sources, respectively (Reed et al. 2003). Expression and purification of *Pf*FEN349 followed the same protocols as described previously for *Pf*FEN405 with the only difference being that for ^2^H,^15^N,^13^C-labelled *Pf*FEN349, the cultures were incubated with shaking for a further 24 h after induction with 0.5 mM IPTG to allow for protein expression. The nuclease activity of *Pf*FEN349 was confirmed by FRET assay (AlMalki et al. 2016). For ^2^H,^15^N,^13^C-labelled *Pf*FEN349, no procedure was necessary to promote back exchange of amide deuterium atoms to amide protium atoms as exposure to protiated solutions during the purification steps facilitated this process. All reagents were of analytical grade and the stable isotopically labelled compound ^13^C_6_,^2^H_7_-D-glucose (U–^13^C_6_, 99%; 1,2,3,4,5,6,6-d_7_ 97–98%) was purchased from CortecNet (France) and used as received.

### NMR spectroscopy of *Pf*FEN349

Initially, a 5-mm NMR tube was loaded with 0.5 mM ^15^N-labelled *Pf*FEN349 in NMR buffer supplemented with ^2^H_2_O (10% v/v) for the deuterium lock and 1 mM TSP as a chemical shift reference. A ^1^H–^15^ N TROSY experiment showed that resonances from the nuclease domain of *Pf*FEN405 and *Pf*FEN349 compared well, but that the large number of peaks at random coil chemical shifts observed for *Pf*FEN405 were now absent in *Pf*FEN349 (Supplementary Fig. 1B). Accordingly, a 5-mm NMR tube was loaded with 0.75 mM ^2^H,^15^N,^13^C-labelled *Pf*FEN349 in NMR buffer supplemented with ^2^H_2_O (10% v/v) for the deuterium lock and 1 mM TSP as a chemical shift reference. All experiments were recorded at 298 K using an 800 MHz Bruker Neo 4-channel spectrometer. Typically, ^1^H–^15^ N TROSY spectra were accumulations of 32 transients with 300 increments and spectra widths of 34 ppm centred at 120 ppm in the indirect ^15^N-dimension. For the backbone ^1^H_N_, ^15^N, ^13^C_α_, ^13^C_β_ and ^13^C′ resonance assignment, standard Bruker pulse sequences for 2D ^1^H–^15^ N TROSY and 3D TROSY-based HNCA, HN(CO)CA, HNCACB, HN(CO)CACB, HN(CA)CO, HNCO, (H)N(COCA)NNH and H(NCOCA)NNH spectra were acquired. The 3D TROSY-based constant time experiments were acquired using non-uniform sampling (NUS) employing a multi-dimensional Poisson Gap scheduling strategy with exponential weighting (Hyberts et al. 2013) at 24% sampling apart from the HNCA, HNCACB, HN(CO)CACB and HN(CA)CO experiments which used 48% sampling. NUS data were reconstructed using either multi-dimensional decomposition or compressed sensing within TopSpin version 4.0.5 (Hyberts et al. 2012). ^1^H chemical shifts were referenced relative to the internal TSP signal resonating at 0.0 ppm, whereas ^15^N and ^13^C chemical shifts were referenced indirectly using nuclei-specific gyromagnetic ratios.

### Extent of assignments and data deposition of *Pf*FEN349

Backbone ^1^H_N_, ^15^N, ^13^C_α_, ^13^C_β_ and ^13^C′ chemical shifts were assigned for substrate-free *Pf*FEN349 using standard triple resonance methodology (Gardner and Kay 1998). Spectra were processed with TopSpin software version 4.0.5 and peak picking was performed using FELIX (Felix NMR, Inc.). Frequency matching of the backbone assignments was achieved using a simulated annealing algorithm employed by the *asstools* assignment program (Reed et al. 2003). The backbone ^1^H_N_, ^15^N, ^13^C_α_, ^13^C_β_ and ^13^C′ chemical shifts have been deposited in the Biological Magnetic Resonance Data Bank (https://www.bmrb.wisc.edu) under the BMRB accession code 53083. 

**Fig. 2:**
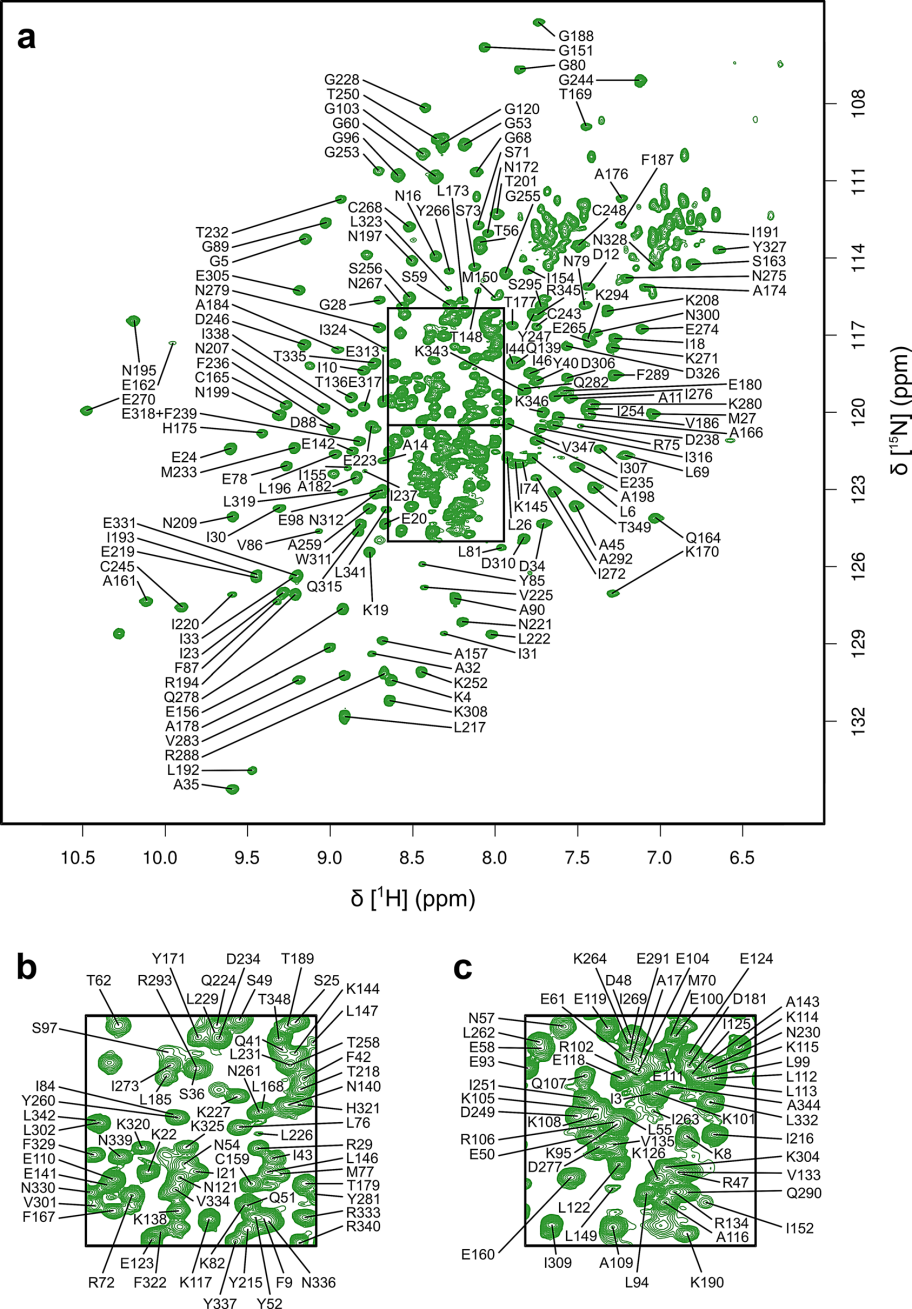
^1^H–^15^N TROSY spectrum of *Pf*FEN349 recorded at pH 7.4 and 298 K. (**a**) The full spectrum is shown, together with 2-fold scale expansions of (**b**) the upper crowded region and (**c**) the lower crowded region. The assignments of the backbone amide resonances are indicated by one-letter residue type and sequence number

Excluding the 10 proline residues along with the N-terminal Gly2 residue, 298 residues out of a total of 337 residues were assigned in the ^1^H–^15^ N TROSY spectrum of *Pf*FEN349 (Fig. 2). In total, 89.9% of all backbone resonances were assigned (^1^H_N_ = 88.4%, ^15^N = 88.4%, ^13^C_α_ = 91.4%, ^13^C_β_ = 89.4% and ^13^C′ = 90.8%). There are 39 residues that remain unassigned in the ^1^H–^15^ N TROSY spectrum: Thr7, Ala13, Met37, Ser38, Leu39, Thr63, Ser64, His65, Ile66, Ser67, Lys127, Gln128, Ser129, Gly130, Arg131, Thr132, Arg137, Asp183, Ala200, Ser202, Asn203, Gln204, Asn205, Lys206, Asn210, Ser211, Lys212, Arg213, Gly214, Cys240, Ile241, Leu242, Lys257, Ser285, Asn286, Phe287, Phe296, Ile297 and Asn298 (Fig. 1B). Using an AlphaFold model of *Pf*FEN349 superposed with the crystal structure of DNA-bound hFEN1 (PDB 3Q8K, Tsutakawa et al. 2011), many of the unassigned residues locate to regions of the enzyme important for binding the DNA substrate and catalysing the chemical step. Residues Thr7 and Met37–Leu39 are positioned within the active site, Asp183 coordinates one of the catalytic magnesium ions and Cys240–Leu242 adopt an α-helical cap that is likely involved in potassium ion coordination. Residues Thr63–Ser67 form an α-helical cap that is close to the DNA substrate, Lys127–Thr132 are located in the helical arch close to the saddle and the Ala200–Gly214 loop and residue Lys257 are also positioned close to the DNA substrate. These residues in substrate-free *Pf*FEN349 have predicted roles in ion binding and DNA accommodation and are involved in conformational exchange dynamics occurring on the millisecond timescale, as is typically observed in substrate-free enzymes (Boehr et al. 2006). The remaining residues (Ala13, Arg137, Ser285–Phe287 and Phe296–Asn298) are located in loops or secondary structure motifs that are solvent exposed and it is likely that their ^1^H–^15^ N TROSY correlations are attenuated by exchange with solvent. Despite the absence of observable peaks for some residues likely involved in DNA binding and catalysis, the presence of assigned residues in the periphery of the active site provides effective reporters for ligand binding. Additionally, such binding events could change the dynamic properties of these unassigned residues allowing their observation in the spectra. Moreover, as active site residues tend to be conserved between species, the search for selective inhibitors at allosteric binding sites would offer the most promise in successful ligand screening studies.

**Fig. 3:**
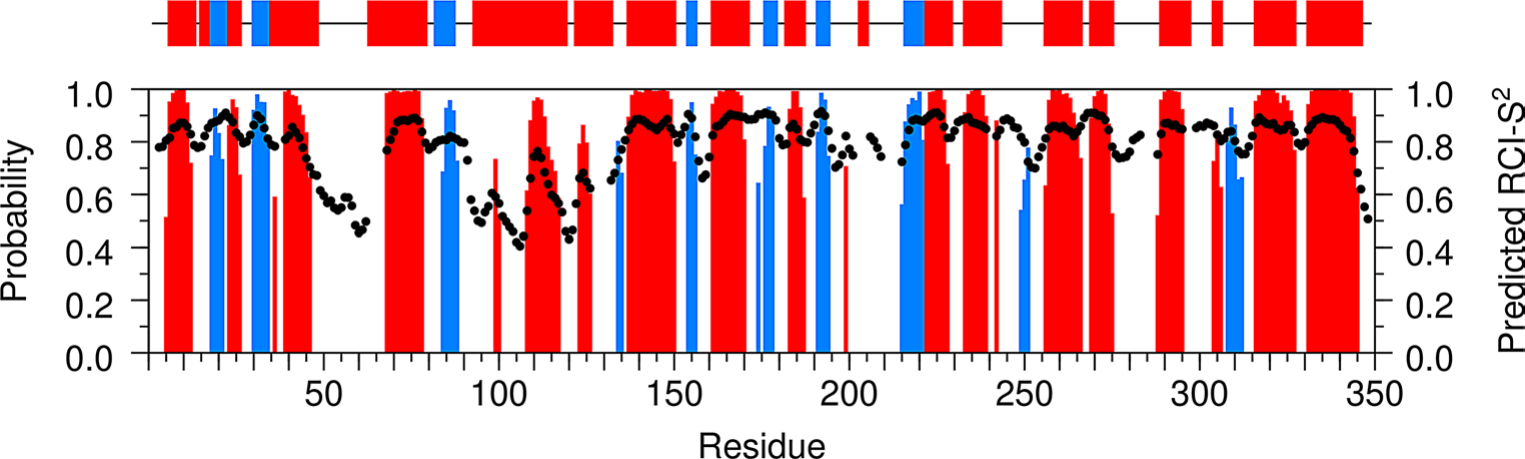
Comparison of secondary structure content derived from the solution behaviour of *Pf*FEN349 and an AlphaFold model of *Pf*FEN349. Predicted secondary structure content and residue-specific random coil index order parameters (RCI-S^2^) of *Pf*FEN349 (lower panel) were obtained with the TALOS-N webserver (Shen and Bax 2013) using the backbone ^1^H_N_, ^15^N, ^13^C_α_, ^13^C_β_ and ^13^C′ chemical shifts. The predicted secondary structure elements are illustrated as red bars for α-helices and blue bars for β-strands, with the height of the bars representing the probability assigned by the software. The predicted RCI-S^2^ values are indicated by black circles. Secondary structure motifs present in an AlphaFold model of *Pf*FEN349 (upper panel) are shown using the same colour representation for α-helices and β-strands

Predictions of secondary structure content and residue-specific random coil index order parameters (RCIS^2^) of *Pf*FEN349 were obtained by uploading the backbone ^1^H_N_, ^15^N, ^13^C_α_, ^13^C_β_ and ^13^C′ assigned chemical shifts to the TALOS-N webserver (Shen and Bax 2013). The predicted secondary structure for the solution conformation of *Pf*FEN349 compares well with the secondary structure elements present in an AlphaFold model of *Pf*FEN349 (Fig. 3), thereby validating the predicted model. Furthermore, residues with the highest values of RCIS^2^ are located in well-defined secondary structure motifs, whereas residues with the lowest values correspond to loop regions. However, a few differences can be observed mainly in the helical arch (Glu93–Val133) where the NMR data reveal it to be more disordered than the mostly α-helical prediction of the AlphaFold model. Additionally, two short β-strands (Asp249–Ile251 and Lys308–Asn312) are identified for *Pf*FEN349 in solution, whereas these segments are more flexible and extended in the AlphaFold model. Altogether, the data are in good agreement, which indicates that the solution conformation is comparable with the protein structure in the AlphaFold model and provides confidence in the backbone chemical shift assignments of *Pf*FEN349.

## Electronic supplementary material

Below is the link to the electronic supplementary material.

Supplementary Material 1

## Data Availability

The backbone chemical shifts for P*f*FEN349 have been deposited in the Biological Magnetic Resonance Data Bank (https://www.bmrb.wisc.edu) under the BMRB accession code 53083.
