# Supplementary material for: 1H, 15N and 13C backbone resonance assignments of flap endonuclease from Plasmodium falciparum
Source: Biomol NMR Assign. 2025 Aug 9;19(2):235–43. doi: 10.1007/s12104-025-10241-6 (PMC12513906; doi:10.1007/s12104-025-10241-6)
Supplement: Supplementary file 1 — Supplementary Material 1 [file 12104_2025_10241_MOESM1_ESM.pdf]

Supplementary Information

^{1}H, ^{15}N and ^{13}C backbone resonance assignments of flap endonuclease from *Plasmodium falciparum*

Rodolpho do Aido-Machado[1], Nicola J. Baxter[2,3], Michelle L. Rowe[2], Manoj B. Pohare[1], Srdjan Vitovski[1], Jon R. Sayers[1], Jonathan P. Waltho[2,3,]*

[1] Division of Clinical Medicine, School of Medicine and Population Health, The University of Sheffield, Medical School, Beech Hill Road, Sheffield S10 2RX, UK

[2] School of Biosciences, The University of Sheffield, Firth Court, Western Bank, Sheffield, S10 2TN, UK

[3] Manchester Institute of Biotechnology and School of Chemistry, The University of Manchester, 131 Princess Street, Manchester, M1 7DN, UK

* Corresponding author: Jonathan P. Waltho, email: j.waltho@sheffield.ac.uk

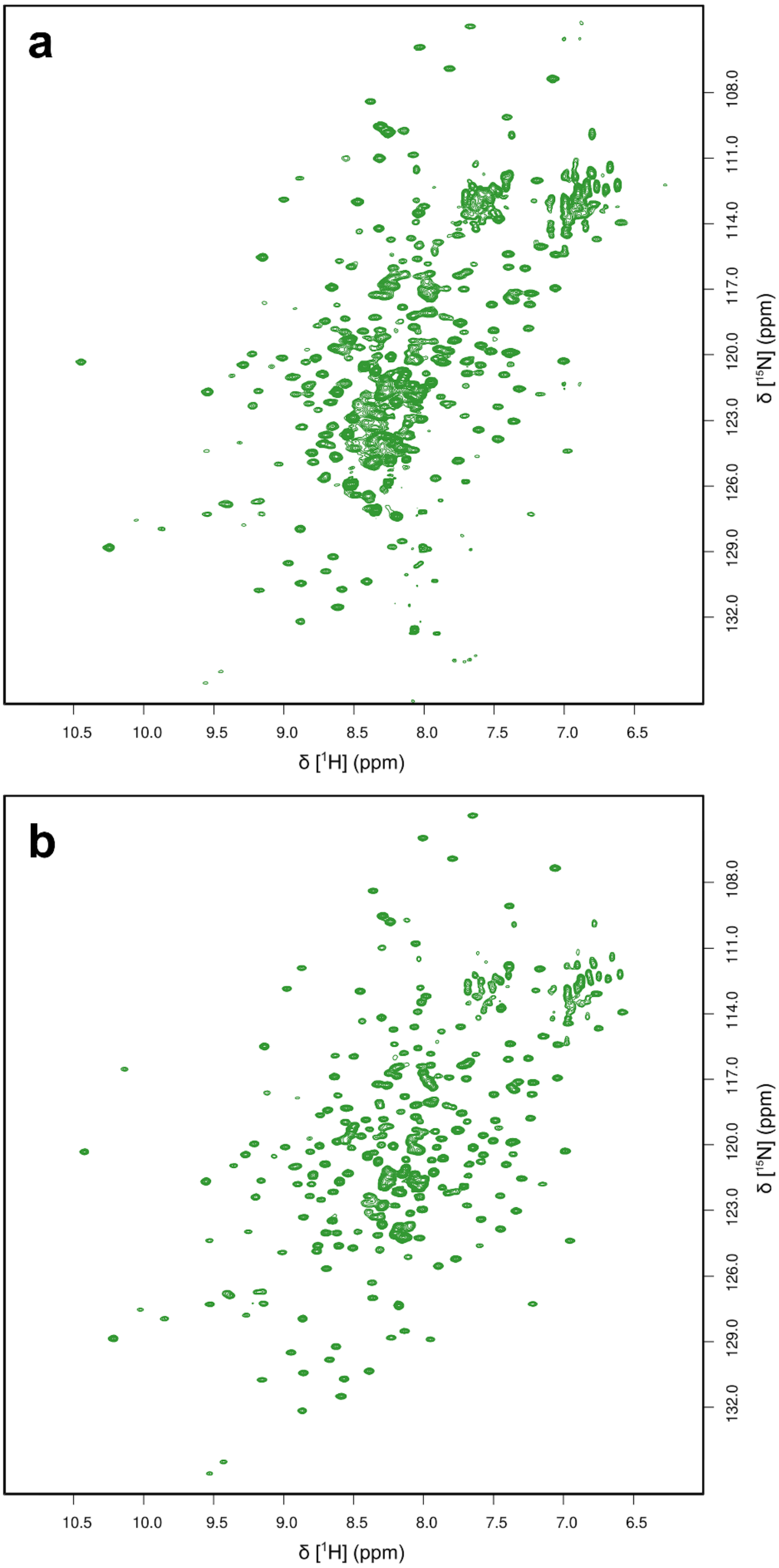

Supplementary Fig. 1 Comparison of the solution behaviour of *Pf*FEN405 and *Pf*FEN349 recorded at pH 7.4 and 298 K. (a) A ^{1}H–^{15}N TROSY spectrum of ^{15}N-labelled *Pf*FEN405. **(b)** A ^{1}H–^{15}N TROSY spectrum of ^{15}N-labelled *Pf*FEN349. The spectra show that the N-terminal nuclease domain of *Pf*FEN405 and *Pf*FEN349 compare well and are folded under the conditions of the NMR experiment. The large number of peaks at random coil chemical shifts observed for *Pf*FEN405 are now absent in *Pf*FEN349 following removal of the disordered residues located in the C-terminal domain
